# Exoscopic extradural anterior clinoidectomy

**DOI:** 10.3171/2023.10.FOCVID23118

**Published:** 2024-01-01

**Authors:** Anna Maria Auricchio, Francesco Calvanese, Martin Lehecka

**Affiliations:** 1 Department of Neurosurgery, Helsinki University Hospital and University of Helsinki, Finland; and; 2 Department of Neurosurgery, University Hospital Foundation A. Gemelli IRCCS, Catholic University of the Sacred Heart, Rome, Italy

**Keywords:** exoscope, extradural anterior clinoidectomy, clinoidal meningioma, drilling, optic nerve decompression

## Abstract

Extradural anterior clinoidectomy is a resourceful technique to decompress the optic nerve as well as increase exposure of the parasellar region during extensive approaches. Despite requiring adjunctive epidural bone work, this technique allows safe optic nerve mobilization and early devascularization for anterior clinoidal meningioma resection. This 2D operative video describes right optic nerve decompression by extradural anterior clinoidectomy and subsequent resection of a right Al-Mefty type III clinoid meningioma under exoscope magnification. The patient was a 50-year-old woman with a 1-year history of right visual acuity impairment and papillary atrophy. The exoscope allows a 360° view around the anterior clinoid, improving maneuverability.

The video can be found here: https://stream.cadmore.media/r10.3171/2023.10.FOCVID23118

## Transcript

This 2D operative video shows a right optic nerve decompression by extradural anterior clinoidectomy and subsequent resection of a small clinoid meningioma (Al-Mefty type III) using an exoscope (Aesculap AEOS).

### 0:40 Clinical History.

The patient is a 50-year-old woman with a 12-month history of progressive visual acuity impairment and papillary atrophy in the right eye, confirmed by ophthalmological examination. No other neurological symptoms were reported. The MRI shows a right-sided anterior clinoid meningioma, classified as Al-Mefty type III. The tumor is rather small, but it is located just at the orifice of the optic canal, as seen on T1-weighted images. On the T2 images, we can see that the tumor is pushing the right optic nerve laterally, and it is growing into the optic canal, compressing the nerve at this level.

### 1:15 Surgical Planning.

Considering the progressive visual worsening, we decided to decompress the right optic nerve by opening of the bony optic canal and removing the intradural part of the tumor through a mini-pterional approach combined with extradural anterior clinoidectomy. The extradural approach is used to gain access to the optic canal along its whole length all the way to the orbital apex.

### 1:36 Patient Positioning and Craniotomy.

Patient’s head is fixed in a Sugita head frame, rotated 50°–60° to the contralateral side and slightly extended. After minimal shaving, a curvilinear skin incision is planned just behind the hairline from the midline toward the right ear up to 3 cm cranial to the origin of the zygoma. After submuscular dissection, a Helsinki-style mini-pterional approach is used with the whole bone flap placed under the temporal muscle. Medial and lateral craniotome cuts are connected by a groove along the frontobasal part crossing the sphenoid ridge. The bone flap of 3–4 cm is then cracked along this groove and elevated.

### 2:12 Anterior Clinoid Anatomy.

A 3D CTA reconstruction shows the surgical steps during drilling of the bony segments needed to reach the full removal of the anterior clinoid process. The blue area represents the sphenoid ridge that has to be drilled first to reach the medial clinoidal structures, consisting of the superior and inferior roots. The superior clinoidal root (light green area) connects the anterior clinoid process to the planum sphenoidale and forms the roof of the optic canal, together with the falciform ligament. The inferior clinoidal root (red area) consists of the optic strut that connects the clinoid to the sphenoid body, resulting in an inferolateral wall of the optic canal. This structure also divides the optic canal from the superior orbital fissure. The 3D rotational view shows the relationship of the anterior clinoid process, the optic canal, and the superior orbital fissure. It also shows the relationship with the cavernous and clinoidal segments of the internal carotid artery (ICA), the C4 and the C5 segments according to Bouthillier classification.

### 3:16 Drilling of the Sphenoid Ridge.

The OR setting, exoscope position, and surgeon ergonomics are shown here.

After flattening of the pterion, the sphenoid ridge is progressively drilled in the proximal direction with a 5-mm coarse diamond.

The orbito-meningeal artery is exposed, coagulated, and cut.

The drilling is continued medially with a 4- and 3-mm fine diamond, until the meningo-orbital band. The meningo-orbital band is cut, allowing for detachment of the dura of the temporal fossa from the lateral wall of the cavernous sinus as shown by the dotted line and orange arrows. This step exposes the clinoidal body highlighted in blue.

### 4:14 Drilling of the Superior Root and Optic Canal Unroofing.

The drilling is then shifted more medially on the most superior part of the anterior clinoid process (light green area), with the aim to drill along the optic nerve starting from lateral to medial and posterior to anterior. Drilling is done under higher magnification to have better control over the underlying structures.

The expected location of the optic nerve is shown in violet color, the residual superior root in yellow, the optic strut in red, and the tip of the clinoidal process in green. The drilling is continued with fine 2-mm diamond until only a translucent sheet of bone remains on top of the optic nerve. This bony shell is then removed with a microdissector. Now the clinoidal body is free from the planum sphenoidale and the superior part of the optic nerve is already decompressed.

### 5:17 Drilling of the Optic Strut.

Next aim is to decompress also the lateral wall of the optic canal by drilling the optic strut shown in red color next to the optic nerve shown in violet. The fine adjustments of the exoscope camera angulation using a foot pedal is very helpful to gain optimal view for drilling. With this drilling completed, the optic canal is already decompressed for nearly 180° and the tip of the anterior clinoid process (shown in dark green) is free from its bony attachments.

### 5:55 Drilling of the Clinoidal Body.

What is left is to remove the tip of the anterior clinoid process as shown in light green color. This is achieved by first cavitating the bone from inside and then mobilizing the thin crust from its adhesions to the dura. Extra care is needed not to injure the internal carotid artery lying underneath. Removal of the remaining bone piece leaves the space laterally to the optic nerve completely decompressed.

The graphic shows the dura covering of the right optic nerve in violet, remnant of the superior clinoidal root in yellow, remnant of the optic strut in red, and course of the cavernous segment of the internal carotid artery in pink. The fold of the falciform ligament is free and nicely visualized.

### 6:50 Intradural Steps and Tumor Resection.

With the extradural work finished, it is time to move intradurally.

The dura is opened in a curvilinear fashion showing the sylvian veins, the frontal and temporal lobe. We move subfrontally and immediately reach the attachment of the meningioma at the frontobasal dura next to the optic nerve. Laterally, we identify supraclinoidal ICA and the lateral part of the optic nerve, which is compressed by the tumor.

Using bipolar forceps and suction, the tumor is first detached from its origin and then removed in several pieces to decompress the optic nerve.

### 7:36 Cutting the Falciform Ligament.

After removal of the exophytic intradural part of the meningioma, what is left of the tumor is the part growing into the optic canal. It is usually at the level of the falciform ligament where the optic nerve is compressed the most. At this stage of the surgery, due to the previous extradural optic canal decompression, there is already some extra space. Still, to remove the remaining part of the tumor, the falciform ligament is cut along the optic nerve and the optic nerve is decompressed further. The cutting of the falciform ligament can be performed using either microscissor or with an angled knife. We can see how the optic nerve has slightly darker color at the site where the maximal compression was at the level of the falciform ligament. The small tumor remnants are gently dissected away from the nerve, sparing its vascularization.

The course of the ophthalmic artery is usually inferolaterally to the optic nerve at the beginning of the optic canal, as in this case, so dissecting above and medially to the nerve, with a blunt hook was relatively safe.

The optic nerve is now free, and we can also see the intradural course of the ICA, the origin of the posterior communicating artery, as well as the right-sided olfactory nerve.

### 9:15 Closure.

Before the final closure, we use the possibility to inspect the most distal part of the optic canal from extradural direction for any tumor remnants. For this, the dural cut along the nerve is enlarged a little. At the start of closure, a thin sheet of Gelfoam is inserted intradurally. Autologous fat is then used to fill the epidural space and cover the small dural defect.

The dura is sutured in a watertight fashion. Bone flap is attached with two CranioFixes and rest of the closure is carried in layers.

### 9:58 Clinical Outcome.

Patient was discharged on the 3rd postoperative day. At 3-month ophthalmological follow-up, the findings were the same as before the surgery. There had been no further progression of the symptoms of the right eye. Unfortunately, there was no major improvement as the preoperative compression damage to the nerve had probably already been irreversible.

### 10:18 References.

Other papers discussing this topic.^1–5^

## Disclosures

The authors report no conflict of interest concerning the materials or methods used in this study or the findings specified in this publication.

## Author Contributions

Primary surgeon: Lehecka. Assistant surgeon: Auricchio, Calvanese. Editing and drafting the video and abstract: all authors. Critically revising the work: all authors. Reviewed submitted version of the work: all authors. Approved the final version of the work on behalf of all authors: Calvanese. Supervision: Lehecka.

## Supplemental Information

### Patient Informed Consent

The necessary patient informed consent was obtained in this study.
